# Microbiome Multi-Omics Network Analysis: Statistical Considerations, Limitations, and Opportunities

**DOI:** 10.3389/fgene.2019.00995

**Published:** 2019-11-08

**Authors:** Duo Jiang, Courtney R. Armour, Chenxiao Hu, Meng Mei, Chuan Tian, Thomas J. Sharpton, Yuan Jiang

**Affiliations:** ^1^Department of Statistics, Oregon State University, Corvallis, OR, United States; ^2^Department of Microbiology, Oregon State University, Corvallis, OR, United States

**Keywords:** compositionality, heterogeneity, microbiome networks, multi-omics data integration, network analysis, normalization, sparsity

## Abstract

The advent of large-scale microbiome studies affords newfound analytical opportunities to understand how these communities of microbes operate and relate to their environment. However, the analytical methodology needed to model microbiome data and integrate them with other data constructs remains nascent. This emergent analytical toolset frequently ports over techniques developed in other multi-omics investigations, especially the growing array of statistical and computational techniques for integrating and representing data through networks. While network analysis has emerged as a powerful approach to modeling microbiome data, oftentimes by integrating these data with other types of omics data to discern their functional linkages, it is not always evident if the statistical details of the approach being applied are consistent with the assumptions of microbiome data or how they impact data interpretation. In this review, we overview some of the most important network methods for integrative analysis, with an emphasis on methods that have been applied or have great potential to be applied to the analysis of multi-omics integration of microbiome data. We compare advantages and disadvantages of various statistical tools, assess their applicability to microbiome data, and discuss their biological interpretability. We also highlight on-going statistical challenges and opportunities for integrative network analysis of microbiome data.

## Introduction

The microbiological sciences have undergone a research transformation in recent years as extensive volumes of microbiome data have been generated. By coupling environmental DNA sequencing procedures with bioinformatic and data analytic approaches, scientists have begun to disentangle the composition, diversity, and function of microbiomes (The Human Microbiome Project Consortium, 2012; Sunagawa et al., 2015; Thompson et al., 2017). However, the complexity of microbial systems, which frequently include diverse taxa and ecological covariates, continues to challenge the discovery of biological signal in these massive data sets. One common goal is to resolve how the microbiome influences or responds to its environment (Alivisatos et al., 2015; Blaser et al., 2016). To disentangle these mechanisms among the complex milieu of microbiome features, researchers have developed a rich array of analytical procedures, with one of the most widely used being microbiome network reconstruction.

Networks can be used to itemize interactions between community members, between communities, and between community members and some set of covariates (Follows et al., 2007; Faust et al., 2012; Gaulke et al., 2016; Tapio et al., 2017; Gould et al., 2018; Mandakovic et al., 2018). As a result, they offer a mapping of how information flows among the members of the microbiome or its environment (Röttjers and Faust, 2018). These networks have been most widely applied to microbiome taxonomic data and are traditionally assembled by correlating microbiome features and establishing linkages between features based on the significance or magnitudes of these correlations (Faust and Raes, 2012; Röttjers and Faust, 2018). Networks can then be visualized or analyzed using a variety of techniques to resolve, for example, taxa that potentially co-depend on one another, taxa that potentially compete with one another, or keystone taxa (Faust and Raes, 2012; Layeghifard et al., 2017). More analytically rigorous methods for inferring these taxonomic interactions have recently been developed to resolve the biologically relevant interactions and to account for unique statistical features of microbiome data (Dohlman and Shen, 2019).

While the analysis of networks representing microbe-microbe interactions has transformed our knowledge of how uncultured microbes potentially interact with one another in their environment, a small but growing number of studies increasingly leverage multi-omics networks to infer how microbial taxa interact with features of their environment (Kint et al., 2010; McHardy et al., 2013; Theriot et al., 2014; Morgun et al., 2015; Heintz-Buschart et al., 2016; Pfalzer et al., 2016; Maier et al., 2017). Microbiome multi-omics data involve collecting multiple types of high-dimensional biological data—including 16S, metagenomic, metatranscriptomic, metabolomics, etc.—from a microbiome sample and its environment or host. While these approaches often remain relatively expensive, technological transformations continue to reduce the cost of generating diverse data constructs, which, in turn, increases the rate at which researchers can apply these multi-omics approaches. This increased accessibility is fortunate, as the integration of multi-omics data holds potential to resolve functional mechanisms of the microbiome (Rodrigues et al., 2018; Wang et al., 2019). For example, these data integrative networks can clarify how changes in the relative abundance of a taxon relates to the expression of genes across a microbial community (i.e., the metatranscriptome), the pool of metabolites, or the phenotype of the microbiome’s host. However, there remain relatively few tools that investigators can rely on to integrate and understand these data.

Multi-omics network integration offers an opportunity to resolve how specific members of the microbiome functionally relate to specific environmental features, which, in turn, helps researchers key in on pathways of information flow that may ultimately transform our ability to manipulate, rescue, or mimic microbiomes. However, their application remains nascent. Most studies in this area thus far apply measures of correlation (such as Spearman’s rank correlation) to resolve microbial taxa that correlate with specific environmental or host features. This approach has specifically been used to clarify how gut microbial abundance relates to the pool of intestinal metabolites (McHardy et al., 2013), discern possible connections between mucosal bacterial abundance and intestinal gene expression in association with inflammatory bowel disease (Morgan et al., 2015), resolve which specific microbes on the human skin may produce metabolites of interest (Bouslimani et al., 2015), and uncover how ocean microbes express transcripts (Aylward et al., 2015). However, this relatively simplistic statistical approach does not necessarily meet the assumptions of microbiome data or address the needs of the problems that arise from such data and may yield inappropriate conclusions.

To promote the innovation of statistical approaches that are more appropriate and specific for microbiome multi-omics network analysis, we present a comprehensive review of the currently available network-based statistical methods and discuss their application to multi-omics data integration. In addition, we consider the unique features of microbiome data and microbiome multi-omics data integration and further explore the reviewed network-based statistical methods in terms of their appropriateness and limitation when applied to microbiome multi-omics data integration. At the end, we conclude with remarks on the major challenges and research opportunities in the innovation of statistical approaches for microbiome multi-omics network analysis.

## Overview of Networks

Network data structures are often complex and involve rich and unusual terminology. In this section, we orient readers to basic concepts and terms associated with network data science, with the goal of improving comprehension of the subsequent discussion of network-based statistical approaches (Section “Review of Available Network-Based Procedures”).

Networks, which are also called graphs, are useful data structures for examining how components of a system interact with or relate to one another. These interactions are commonly derived using statistical approaches that reveal associations between pairs of components and are further illustrated graphically as edges that connect pairs of nodes that represent the components of a system. Networks can also represent empirical interactions between components that have been experimentally validated. However, in the case of microbiome research, limitations in the number of cultured taxa and the complexity of most microbial communities restrict the application of such empirical approaches. Networks have been effectively used in a variety of fields. Examples include infectious disease research (Silk et al., 2017), social interaction analysis applied to marketing (Liu et al., 2019) and political science (Cranmer et al., 2017), analysis of neuroimaging data (Fujita et al., 2017), information flow through the internet (Dorogovtsev and Mendes, 2003), genomics data analysis (Kleaveland et al., 2018). In microbiome science, network data structures have been used in a variety of contexts (as reviewed by Faust and Raes, 2012, and Layeghifard et al., 2017), including efforts to evaluate interactions between members of a microbial community (Faust et al., 2012), associate taxa with metabolite production (Bouslimani et al., 2015), and determine which taxa interact with host bile acid metabolism (Theriot et al., 2016).

Networks adopt a variety of terms and properties, some of which we define here to orient readers. The components of the system being modeled by a network are represented as nodes or vertices. In microbiome research nodes can be biological features such as microbial taxa, genes, metabolites, and proteins. Nodes may also represent environmental or host features, such as pH and markers of immune status. The presence of an edge between a pair of nodes indicates an association between the nodes, such as a correlation between the abundance of two taxa. Such edges may suggest a dependency between the taxa by indicating, for example, that when one taxon increases in abundance, the other taxa do as well possibly due to cross-feeding. We note that an inferred edge itself does not imply a causal dependency between the features, the inference of which requires a controlled experiment. If the associations differ in strength, edges can be weighted to illustrate the strength of association and guide interpretation. The distinction between positive and negative associations can also be captured by weights of different signs. In some cases, the interactions being modeled by a network are directed, meaning that they indicate that the change to one component causes a change in another connected component. In such instances, such directed network edges are represented by arrows and can be used to depict the cause and effect relationships among components. It is worth noting that causality can be challenging or impossible to infer in many genomic investigations depending on the study design. In those cases, the directionality of the relationship might be pre-specified based on knowledge to construct a bipartite network (e.g., in some regression models, see Section “Regression-Based Methods”) or inferred using the data in a probabilistic framework as a way of representing the information propagation in the system (e.g., in Bayesian networks, see Section “Bayesian Networks”).

In this article, our main interest is in the problem of estimating or constructing a network by integrating two or more types of omics data including microbiome data. In the rest of this article, the variables representing the components corresponding to each data type will be referred to as “features.” Variables from different types of omics data will be said to belong to different “feature types.” Examples of feature types include but are not limited to microbiome taxonomic, transcriptomic, and metabolomic features. The corresponding features within these feature types may include the abundance of a microbial taxon, the expression level of a gene, and the concentration of a metabolite. Depending on the scientific question of interest and the analytical approach used, there are various types of networks that can be constructed based on multi-omics data. When considering associations between distinct feature types, a bipartite network can be used where the edges are drawn between nodes of different types (as reviewed by Pavlopoulos et al., 2018). Alternatively, it is possible to construct a network among features of a single type where data from another type are incorporated in the analysis as additional information or covariates to improve the estimation of the network. Examples of this approach include studies conducted by Li et al. (2012) and Chun et al. (2013), a more detailed discussion of which can be found in Section “Methods Based on Graphical Models”.

Once networks are estimated from the data, there are numerous metrics that can be quantified on the networks to summarize the overall structure of the system. One of the primary metrics used is degree, which is the count of edges that connect one node to all the others. Nodes with higher degree represent features that are relatively highly connected to other features in the system being modeled. Such nodes may have more influence on the system’s dynamics and may represent, for example, keystone taxa in a community. Most real-world networks have a right-skewed degree distribution where most vertices have low degree, and few have high degree. When the degree distribution monotonically decreases over its entire range, it has a power-law distribution and is referred to as a scale-free network. In a scale-free network, some nodes can have significantly higher degree than others. Such nodes are often referred to as “hubs” because they are strong participants of the interactions in the network. Another way to identify important nodes is through measures of betweenness. To calculate betweenness, the shortest path between each pair of nodes in the network is first identified. Then, the betweenness for each node is measured as the number of times the node in question lies in the shortest path between two other nodes. Nodes with high betweenness are potentially influential in the network since they come between many pairs of nodes. Nodes with high betweenness can also have high degree; however, that is not always the case. High-betweenness nodes are often interpreted as bottlenecks of the information flow in the network. Various other topological properties of the network can also be assessed to glean interesting biological insights into a system, such as modularity, which aims to identify clusters of nodes densely connected to each other, with relatively low connectivity to the rest of the network. We refer the readers to the papers of Newman (2010), Ma’ayan (2011), and Charitou et al. (2016) for more in-depth discussions of topological analysis techniques. In this review, we will focus on the statistical estimation of networks instead of the topological analysis of an estimated network.

## Review of Available Network-Based Procedures

In recent years, integrative network analysis has increased in popularity, particularly for multi-omics data sets. The statistical methods utilized in these analyses lend perspective to how microbiome multi-omics networks can be inferred. In this section, we review network-based statistical methods with an emphasis on their applications to multi-omics data integration. We categorize commonly adopted methods into six types and present a detailed review of each type. **Table 1** provides a summary and a comparison of the six types of methods alongside software packages that enable their implementation.

**Table 1:** Summary of available network-based procedures.

Method type	Network type	Representative methods (software: packages)	Advantages	Disadvantages
Marginal correlation analysis	Undirected	Pearson’s correlation, Spearman’s rank correlation, Kendall’s tau (R: base); Local similarity analysis (Linux: ELSA); WGCNA (R: WGCNA)	Easy to implement; nonparametric options available.	Subject to spurious findings due to confounding.
Dimension reduction methods	Typically undirected	PCA (R: base); CCA (R: CCA); PLS (R: pls); CIA (R: ade4); Sparse CCA, Sparse multiple CCA (R: PMA); Sparse PLS (R: spls); Sparse CIA (R: pCIA); Kernel PCA, kernel CCA (R: kernlab)	Can be used to construct networks linking modules of features.	Poor interpretability because each node represents multiple, if not all, features.
Regression-based methods	Directed or undirected	Linear and generalized linear models (R: base); Linear and generalized linear mixed models (R: nlme, lme4); Regularized regression: Lasso, ridge, elastic net (R: glmnet), SCAD, MCP (R: ncvreg), Group lasso, group elastic net, group SCAD, group MCP (R: grpreg); Regularized multivariate regression: Graph-guided fused lasso (R: GFLASSO), remMap (R: remMap), Reduced-rank regression (R: rrpack)	Easy to incorporate covariates; a large number of statistical methods and software tools are available.	Need to specify each feature as either a response variable or a predictor.
Graphical models	Undirected	Graphical lasso (R: glasso, huge); Neighbourhood selection (R: huge); Joint graphical lasso (R: JGL); Conditional graphical models Covariated-adjusted graphical models (R code: caPC)	Conditional dependency captures direct biological interactions more effectively than methods based on marginal correlations.	Most methods assume a multivariate normal distribution.
Bayesian networks	Directed	CONEXIC (Linux: CONEXIC); QTLnet (R: qtlnet); Bayesian Network Prior (MATLAB: BNP); Search-and-score approaches, constrain-based approaches (R: bnlearn)	Links more directly related to causality; ability to incorporate prior knowledge; possibility to handle data following disparate distribution types.	Current methods do not scale well to massive data sets.
Network integration	Undirected	GeneMania (Cytoscape/Web: GeneMANIA); SNF (R: SNPtools); DCA (MATLAB: Mathup)	Often simple to implement; ability to borrow information from multiple networks.	Individual networks that serve as the input of the methods must be reliably estimated; a shared biological mechanism is assumed.

### Marginal Correlation Analysis

The most commonly applied statistical method for constructing biological networks is marginal correlation analysis. In this analysis, the relationship between two biological features, such as genes, transcripts, proteins, metabolites, and microbes, is described by the correlation of their expression, concentration, or abundance levels inferred from multiple statistically independent observations, such as biological replicates or samples. Technically, this relationship can be quantified by any statistical measure of correlation, including but not limited to Pearson’s correlation, Spearman’s rank correlation, and Kendall’s tau, as long as the approach is meaningful for a given biological context. Marginal correlation analysis is also useful when integrating multiple biological feature types (e.g., genes, transcripts, and proteins) to uncover relationships across feature types (Heintz-Buschart et al., 2016; Bakker et al., 2018; Frost and Amos 2018; McGrail et al., 2018).

Marginal correlation analysis can also be extended to observations that are statistically dependent. For example, consider the case wherein two biological features are observed over time (i.e., two time series of measures). One might want to assess the correlation of the features across the time series. In this case, it is essential that the correlation measures account for the longitudinal nature of the observations. One approach to this problem is the so-called local similarity analysis of two time series (Ruan et al., 2006). In this approach, both time series are first transformed separately to their normal scores. Then, for any subsequence of the first time series starting from the beginning, all subsequences of the same length from the second time series are identified within some predefined time delay. Pearson’s correlations are then calculated between each pair of subsequences across the two time series. Finally, the local similarity score is defined as the maximum correlation for all such possible pairs of subsequences, aiming to find associations with possible delays between the two time series. Local similarity analysis has proven useful for detecting co-varying pairs of microbes as well as the association between a microbe and an environmental factor (e.g., temperature), especially when the variations between features are not synchronous (Ruan et al., 2006).

While the abovementioned methods are purely data-driven, other methods construct biological networks based on both statistical correlations and existing biological knowledge. For example, to create a bipartite network describing the relationship between mRNAs and miRNAs, Gade et al. (2011) combined two p-values for each pair of mRNA and miRNA expression values: (a) a p-value measuring the statistical correlation of the observed data and (b) a p-value obtained from an existing database of miRNA-target predictions (e.g., miRBase) (Griffiths-Jones et al., 2008). The authors applied a truncated product method of combining p-values (Zaykin et al., 2002), which they then transformed to weights and viewed as the adjacency matrix of a bipartite network describing the relationship between mRNAs and miRNAs.

In order to produce a biological network that facilitates meaningful interpretations, studies often only include correlations in the network that manifest correlation coefficients whose absolute value exceeds a threshold, which is usually arbitrarily determined, or if its associated p-value is less than a significance level such as 0.05. In the latter case, some applications simply use the raw p-values, which tend to yield excessive false positive edges, while other applications more carefully control false positives by adjusting the p-values with a multiple testing correction for familywise error rate (FWER) or false discovery rate (FDR). A biological network is then constructed by connecting those pairs of biological features with a statistically robust correlation and leaving all other pairs unconnected.

The abovementioned thresholding procedure produces a biological network that is unweighted, in the sense that an edge either exists or not between any pair of nodes. Weighted networks based on marginal correlation analysis have also attracted recent attention, such as in the case of Weighted Gene Co-expression Network Analysis (WGCNA) (Zhang and Horvath, 2005; Langfelder and Horvath, 2008). In this method, an edge in a network is weighted by a soft thresholding function of the inferred correlation (e.g., the sigmoid function, the power adjacency function, etc.) on a continuous scale. Many topological analysis methods have also been extended from unweighted networks to weighted networks, such as node connectivity (Barrat et al., 2003; Amano et al., 2018), network modules (Newman, 2004; Li et al., 2011; Lecca and Re, 2015), clustering coefficient (Opsahl and Panzarasa, 2009), and scale-free topology (Tan and Lei, 2013; Zhang et al., 2015). Because weighted networks encode additional information in the form of connection strengths as compared to unweighted networks, weighted networks have been shown to be a useful option for many biological datasets, including but not limited to microarray data (Kadarmideen et al., 2011; Mohammadnejad et al., 2019), single cell RNA-Seq data (Xue et al., 2013), DNA methylation data (Horvath et al., 2012; Wang et al., 2016), and microbiome data (Tong et al., 2013; Li et al., 2019).

Marginal correlation analysis is probably the most commonly used method to infer biological networks due to its computational simplicity. However, the approach is limited by the fact that it can only infer relationships between pairs of biological features and does not consider how the observed relationship may depend upon other variables or features. As a result, marginal correlation analysis can lead to spurious correlations: two features that independently interact with a third, but not with one another, may appear to correlate. Therefore, marginal correlation analysis is known to be prone to false positives when seeking to identify direct interactions or causal effects among the features. It is important to keep this limitation in mind and to critically assess the risk of confounding factors before drawing conclusions about biological interactions that result from marginal correlation analysis.

### Dimension Reduction Methods

Dimension reduction, such as the widely used method principal component analysis (PCA), is a useful statistical tool that aims to reduce the dimension of a set of variables while retaining as much information from the original data as possible. It is also useful when the relationships between two feature types are investigated, in which case data associated with each feature type are reduced to a lower dimension in a way that captures as much association between the two feature types as possible. We refer the readers to the review papers of Burges (2009) and Engel et al. (2011) as two statistical reviews on dimension reduction and to the review paper of Meng et al. (2016) as a review on the application of dimension reduction to the integrative analysis of multi-omics data.

Commonly used dimension reduction tools include canonical correlation analysis (CCA), partial least square regression (PLS), and co-inertia analysis (CIA) (Meng et al., 2016). These tools share the same goal of summarizing the variables in each feature type by using a small number of linear combinations so as to maximize the association between the two feature types as demonstrated by these linear combinations. Different measures of association correspond to different tools in this category. More specifically, CCA uses Pearson’s correlation to capture the association between two linear combinations (or equivalently, all linear combinations are normalized to have a unit variance), PLS uses covariance to quantify the association with the constraint that the linear combination from one feature type has a unit variance, and CIA uses covariance to represent the similarity with no variance constraint. CCA, PLS, and CIA have all been applied to infer biological networks from multi-omics data. For example, CCA was used to construct gene co-expression networks by considering linear combinations of gene expression at the exon or base pair level for each gene obtained from an RNA-seq dataset (Hong et al., 2013). In this study, the authors then calculated the canonical correlation between each pair of genes, ranked the correlations based on their magnitude, and constructed a co-expression network by retaining a predetermined percentage of edges. In other studies, CIA was applied to mRNA and microRNA data to determine which microRNAs regulates gene expressions (Jovanović et al., 2014) as well as to microbiome and metabolomic data sets to understand the impact of a short-term increase in dietary fiber intake on the gut microbial community (Tap et al., 2015). PLS has also been utilized in multi-omics studies, for example, to analyze the associations between biomarkers for insulin sensitivity and a variety of omic data, including gut microbiota, adipose gene expression, and metabolomic data (Dao et al., 2019).

These methods suffer from a few limitations, which recent efforts have sought to overcome. The first limitation stems from the fact that a linear combination found by CCA, PLS, and CIA tends to include every variable under consideration, albeit with varying weights. This tendency to include every variable results in poor interpretability as it can be difficult to determine which variables contribute to the canonical correlations and which do not. Therefore, a desirable extension is to introduce sparsity to the linear combinations, where the coefficients for variables with less contribution are shrunk to zero. Recent methods that apply such a strategy include sparse canonical correlation analysis (SCCA) (Parkhomenko et al., 2007; Waaijenborg et al., 2008; Parkhomenko et al., 2009; Witten and Tibshirani, 2009; Witten et al., 2009; Hardoon and Shawe-Taylor, 2011; Suo et al., 2017), sparse partial least squares (SPLS) (Lê Cao et al., 2008; Chun and Keleş, 2010; Chung and Keles, 2010; Lee et al., 2011), and sparse co-inertia analysis (SCIA) (Min et al., 2018). These methods try to balance between maximizing the correlation between linear combinations defined for different feature types and minimizing the number of variables included in each linear combination. These methods share the same basic idea of incorporating variable selection techniques, such as lasso and elastic net (Tibshirani, 1996; Zou and Hastie, 2005), into traditional dimension reduction methods. As a result, these methods produce a sparse linear combination for each group of variables, although they each differ in either the problem formulation or computational details. These methods have been used to integrate SNP and gene expression data with the goal of identifying a group of SNPs that explain the variation in gene expression across a group of genes while keeping the group sizes sufficiently small to aid biological interpretation (Parkhomenko et al., 2007; Parkhomenko et al., 2009).

Another limitation of the traditional dimension reduction tools is that they can only consider two feature types, i.e., two groups of variables. Extensions of SCCA have been proposed to accommodate the analysis of multiple groups of variables (Witten and Tibshirani, 2009; Tenenhaus et al., 2014). Meng et al. (2014) proposed the multiple CIA method and used it to integrate transcriptomic, proteomic, and metabolomic data. All of these methods aim to find a linear combination from each group of variables so as to maximize the sum of squared pairwise correlations or the sum of squared covariances between each linear combination and a synthetic axis that is also parametrically optimized.

The third limitation of the traditional dimension reduction tools is that they only replace the original features by their linear combinations. Nonlinear dimension reduction tools have also been proposed to overcome this limitation such as kernel-based dimension reduction methods including kernel principal component analysis (KPCA) (Schölkopf et al.,1997), kernel canonical correlation analysis (KCCA) (Lai and Fyfe, 2003), and kernel fusion methods (Daemen et al., 2009). For example, Reverter et al. (2012) applied KPCA to classify disease types using the kernel principal components estimated from gene expression profiles. Daemen et al. (2009) proposed a kernel fusing method for clinical decision support that transforms multi-omics data into a linear combination of their corresponding kernel matrices and implements a classifier based on the combined result.

A common feature of the aforementioned dimension reduction tools for multi-omics data integration is that they are all based on the integration of two or more types of observed data. They are thus sometimes referred to as data-driven methods. Another class of dimension reduction tools try to integrate the observed data with external knowledge and are therefore called knowledge-driven methods. As an example, Yang et al. (2009) proposed a method called knowledge-based matrix factorization (KMF). In this study, the authors used KMF to build a gene co-expression network based on pairwise correlations between gene expression levels while incorporating existing pathway information from external databases such as Gene Ontology (GO) (Gene Ontology Consortium 2004). To incorporate this external knowledge, KMF finds the best low-rank factorization of the correlation matrix so that it is decomposed into the product of three matrices. The left and right matrices are transpose of each other and they approximate the membership of genes in pathways, while the center matrix captures the relationship between the pathways. This procedure allows KMF to construct a gene-gene correlation network whose structure is consistent with external pathway information while also identifying interactions between the pathways.

In summary, dimension reduction methods look for a combination of the features to represent each feature type while maximizing the correlation or covariance between the resulting combinations. Therefore, dimension reduction methods can be regarded as a multivariate extension of marginal correlation analysis. As a result, these methods are subject to the same pitfall that marginal correlation analysis faces (see Section “Marginal Correlation Analysis”); for example, they may lead to spurious correlations caused by confounding factors. In addition, although sparse versions of dimension reduction methods have been developed, lack of interpretability remains a limitation because each combination includes multiple, if not all, biological features in a group, and thus, the inferred relationships cannot be attributed to a specific pair of features.

### Regression-Based Methods

Network inference in multi-omics data have also been formulated as a regression problem. In this case, a series of regression models are fitted by taking one feature type as the response variable and another type as the predictor variable. Associations identified by these regression models are often interpreted as a directed relationship in which the feature type serving as the predictor is considered to affect or explain the feature type serving as the response. However, this inferred effect does not necessarily demonstrate a cause and effect relationship among the variables. For example, to assess the extent to which mRNA abundance was able to explain protein abundance, Nie et al. (2006b) fitted a linear model for each protein-mRNA pair with the former as the response and the latter as a predictor, incorporating multiple sequence features as additional covariates. For noncontinuous data, generalized linear models such as Poisson regression have also been employed to elucidate interactions between genomic features (Nie et al., 2006a). More recently, Yuan et al. (2018) proposed a regression model that aims to infer gene regulatory networks by incorporating DNA methylation and copy number variation as well as their interactions. Regression-based methods have also been used to integrate other types of multi-omics data. Recent examples include a somatic eQTL analysis using linear regression to model the association between gene expression and the mutation status of linked loci while accounting for various covariates including DNA methylation and gene copy number variation (Zhang et al., 2018). Moore and Hoen (2019) discussed the use of the regression framework to analyze RNA-protein interactions.

As opposed to considering a single predictor at a time, each regression model can also simultaneously include a large number of predictors, possibly from multiple feature types, to identify a set of variables that best predict the response. Typically, in these methods, a feature type of interest is regarded as the response data, with the other feature types regarded as the explanatory data. In each regression model, one feature is taken as the response variable, which is fitted against all variables in the explanatory data as predictors. The resulting high dimensionality leads to an underdetermined regression problem and thereby renders ordinary least squares and maximum likelihood estimation ill-posed. Therefore, variable selection techniques are needed to estimate the model parameters.

Regularized regression, the most representative method being lasso (Tibshirani, 1996), is commonly used for variable selection to overcome these limitations (as reviewed by Bickel and Li, 2006, and by Wu et al., 2019, for its application to multi-omics integration). In this case, a penalty term is incorporated in the usual least squares or maximum likelihood objective function in order to shrink some of the set of parameter estimates to zero, hence inducing sparsity in the regression coefficients. This strategy achieves variable selection and parameter estimation simultaneously. Each coefficient estimated to be nonzero is then represented by an edge in the network between the associated predictor and the response. There have been many applications of this approach to multi-omics studies. For example, Kim et al. (2014) and Yuan et al. (2018) estimated networks between DNA methylation, copy number variation, and gene expression based on a set of regularized linear regressions where separate L1 penalties were imposed on the three feature types. Qin et al. (2014) integrated ChIP seq and transcriptome data to infer gene regulatory networks using a regularization method where the L1 penalty is replaced by L0 and L0.5 penalties.

Another type of regression-based method for integrative network inference uses a technique called multivariate regression (Kim et al., 2009; Peng et al., 2012), which includes a multivariate response (i.e., multiple response variables) in a single model. When a multivariate response is modeled against a set of predictors, the unknown coefficients come in the form of a matrix, where an entry is assigned to relate each response variable to each predictor. Constraints are often imposed either on the sparsity or the rank of this coefficient matrix, or both, to ensure that the model can be fitted despite the limited sample size in comparison to the number of parameters. Applications of this approach to multi-omics data usually combine variables from one feature type, which serves as the multivariate response, while another type of omics features serve as the predictor variables. Like methods based on univariate regression, a directed network can be constructed with edges corresponding to nonzero coefficients. However, unlike univariate methods which involve a large number of separate regression models, multivariate regression only fits one joint model, which allows more realistic modeling and simplified understanding of the biological mechanisms *via* sparsity and rank constraints. For example, Goh et al. (2017) proposed a multivariate regression method, which was used to fit time-course mRNA data for >500 genes against binding information of the target genes for >100 transcription factors. Sparsity and low-rank constraints were imposed to account for the fact that many transcription factors are not related to the genes and the samples are correlated due to the study design.

Regression-based methods are widely used to construct biological networks mainly because they are relatively straightforward to implement. Compared with marginal correlation analysis and dimension reduction methods, regression models have the advantage of being able to incorporate relevant covariate information. A regression framework is also equipped with many well-studied statistical tools to flexibly handle specific analytical needs. For example, random effects can be incorporated to account for inter-sample correlation between samples due to study design (Zhang et al., 2013) and to correct for data heterogeneity due to unobserved confounders (Furlotte et al., 2011). The regression-based approach is also empowered by the recent statistical developments in penalized regression to handle high-dimensional data. However, most regression-based methods entail that each feature (or feature type) is identified as either a response variable or a predictor, which can be a nontrivial choice to make especially when the underlying biology is poorly understood for the system being studied.

### Methods Based on Graphical Models

Gaussian graphical models are widely applied in network analysis (as reviewed by Drton and Maathuis, 2017). Specifically, in a multivariate Gaussian distribution, two variables are statistically independent conditional on all the other variables if and only if the corresponding entry in the inverse covariance matrix of the distribution is zero. Then, to construct a network with each edge representing the conditional dependence between two features given all other features, it is equivalent to identify the nonzero entries of the inverse covariance matrix for the multivariate Gaussian distribution. In reality, the data are often high-dimensional with more variables than samples, which leads to a degenerate sample covariance matrix and makes the estimation of the inverse covariance matrix challenging.

There are two major statistical approaches for estimating the inverse covariance matrix in the high-dimensional Gaussian graphical model: the neighborhood selection method (Meinshausen and Bühlmann, 2006) and the graphical lasso method (Yuan and Lin, 2007; Friedman et al., 2008). Both methods yield a sparse estimator of the inverse covariance matrix, whose nonzero entries can be used to construct a network that denotes the conditional dependency between the variables in the Gaussian graphical model. To apply Gaussian graphical models to the integration of multi-omics data, a naive strategy combines all variables from multiple feature types into one vector, which is assumed to follow a multivariate normal distribution (Shin et al., 2014). However, this approach effectively treats all variables as exchangeable, and, in turn, ignores the potentially important information about their group structure.

One typical application of Gaussian graphical models to multi-omics data is the joint Gaussian graphical model, which simultaneously estimates multiple graphical models under some constraints among them. The constraints are often determined by some prior knowledge for the multiple inverse covariance matrices such as their similarity in magnitudes or sparsity or the membership of nodes in biological pathways (Guo et al., 2011; Danaher et al., 2014; Kim et al., 2017). This idea has been applied to find biological networks from different groups simultaneously, e.g., disease subtypes or experimental conditions. For example, Kim et al. (2017) used a joint Gaussian graphical model to estimate multiple mRNA expression networks from different datasets. Zhang et al. (2016) further extended the idea of joint graphical models to a two-dimensional joint graphical lasso model. This model imposed a joint penalty function to simultaneously estimate two gene expression networks that are patient group-specific from gene expression profiles collected from different data generation platforms. After obtaining the gene networks, the differential networks between the two patient groups were constructed by calculating the differences of dependencies between two group-specific networks (i.e., one differential network for each platform).

Bayesian inference based on joint Gaussian graphical models has also been used to construct networks by applying a G-Wishart prior on the inverse covariance matrix (Peterson et al., 2015). In this particular case, a Markov random field prior was imposed to encourage common edges between joint graph structures. This procedure enabled the identification of which groups have a shared network structure by placing a spike-and-slab prior on parameters which measure network relatedness.

Conditional graphical models represent another class of graphical model approaches that are useful for solving data integration problems. Different from the traditional graphical models, the conditional graphical model incorporates an additional conditioning step to remove spurious dependence that may be caused by common external factors. For example, two genes may depend on each other only because they are regulated by the same DNA markers and have no relationship otherwise. Along this research direction, Li et al. (2012) proposed a method which infers such a conditional graphical model in two steps. It first estimates the conditional covariance matrix and then uses penalized maximum likelihood to obtain the inverse conditional covariance estimator. The authors used their method to define a gene expression network conditional upon eQTL data. Moreover, Chun et al. (2013) extended the same idea to multiple conditional graphical models, allowing the integration of gene expression data from different sources, say, heart and fat tissues. Other similar research includes the covariate-adjusted graphical models that use genetic markers (SNPs) as covariates to correct both false positives and false negatives in gene regulatory networks (Cai et al., 2013; Gao and Cui, 2015). In these methods, the effect of genetic variation is estimated in the first step. Then, the graphical structure is estimated in the second step while adjusting for the genetic effects.

Like graphical lasso, most joint or conditional graphical models incorporate the sparsity assumption to tackle the high dimensionality problem in the context of inverse covariance matrix estimation, but often rely on the assumption of a multivariate Gaussian distribution. Zhang et al. (2017) is one of the few studies that estimate the inverse covariance matrix under a mixed model that includes different biological feature types by accommodating both discrete and continuous variables. Due to the computational complexity of discrete variables, the authors used the pseudo-likelihood method instead of the usual likelihood method for parameter estimation. In spite of these innovations, methods based on graphical models still need to account for the unique characteristics of microbiome data when applied to microbiome multi-omics data integration (Section “Unique Challenges of Microbiome Multi-Omics Network Analysis”).

### Bayesian Networks

Like Gaussian graphical models, Bayesian networks are probabilistic graphical models and are increasingly used as a statistical and machine learning tool for analyzing genomic data. In a Bayesian network, a graph with directed edges is used to represent the conditional relationships in the joint probability distribution of a set of variables: for each variable X, given its parent variables (i.e., nodes pointing to X), X only affects its child variables (i.e., nodes pointed to by X) and is conditionally independent of all other variables. These conditional independence constraints serve to cut down, frequently substantially, the number of parameters needed to jointly model the variables. We refer the readers to a review paper (Koski and Noble, 2014) for a more thorough introduction to Bayesian networks.

In the past decade, Bayesian networks have seen many applications in genomic data integration. For example, Akavia et al. (2010) introduced an algorithm based on Bayesian networks (CONEXIC) to identify driver mutations in cancer by integrating gene expression data with matched copy number data. QTLnet (Chaibub-Neto et al., 2010) is a method that uses a Bayesian network that includes both phenotype and genotype variables as nodes to jointly estimate the causal network between multiple phenotypes and their respective genetic architecture. In order to improve the recovery of gene interaction networks based on experimental data, Isci et al. (2014) proposed a hierarchical method called BNP where a Bayesian network is nested within a classical Bayesian modeling framework. This approach enables the incorporation of rich external knowledge about gene interactions as the prior information in the Bayesian inference procedure. More recently, Khanna et al. (2018) applied Bayesian network to elucidate the interplay between genotype information, neuroimaging measurements, and clinical data to help uncover biological mechanisms underlying Alzheimer’s disease.

The Bayesian network approach has several appealing advantages when applied to multi-omics data analysis. First, because of the structure of the underlying probabilistic model, Bayesian networks are usually considered akin to directed networks, in such that causal relationships are often inferred among nodes. In particular, network edges are often interpreted to represent how information propagates between variables or components in a biological process. We note that, although causal interpretation of Bayesian networks is appealing and widespread, there have been growing skepticism over the liberal use of such interpretation because a Bayesian network does not guarantee causality (Korb and Nicholson, 2008). Second, Bayesian networks can incorporate prior knowledge about plausible relationships among variables within or between feature types (Ni et al., 2014). Third, Bayesian networks may be set up in a way that allows for simultaneous modeling of variables following different types of distributions. For example, Chaibub-Neto et al. (2010) modeled a Bayesian network where the nodes consist of a mixture of continuous phenotype variables and discrete genetic variables. The ability to handle disparate data types is an attractive feature as multi-omics studies frequently involve feature types that are more appropriately modeled using different distributions such as continuous, count, and binary data.

However, a major challenge limiting the use of Bayesian networks in genomic studies is its steep computational cost. The estimation of the structure of a Bayesian network usually involves the optimization of a complicated objective function over a large, nonconvex search space. As the number of variables increases, the computational burden increases super exponentially. Consequently, in most applications of Bayesian networks to multi-omics data, either only a small to moderate number of omics variables are considered or dimension reduction techniques are applied to reduce the number of variables before implementing Bayesian networks.

### Network Integration

A key goal of multi-omics data integration is to create a comprehensive view of a biological process from diverse types of omics data. Network integration approaches seek to solve this problem by integrating multiple, distinct biological networks assembled from different data types. There are many network integration strategies and we review below a representative subset of these approaches.

One approach to this problem, as illustrated by the method GeneMANIA (Mostafavi et al., 2008), is to build a composite association network by taking a weighted average of multiple association networks between features, such as genes, where the weights are selected based upon the composite network’s ability to reconstruct referential characteristics of the features. For example, GeneMANIA uses ridge regression (Hoerl and Kennard, 1970) to find the weights of individual association networks to minimize the difference between the composite network and a target network constructed from known gene functions (such as GO functional categories), while incorporating the prior information of the weights in the ridge penalty.

Diffusion component analysis (DCA) (Cho et al., 2015; Wang et al., 2015; Cho et al., 2016) is another network integration method that targets heterogeneous networks with different connectivity patterns. In DCA, the diffusion state of each node is analyzed with the random walk with restart (RWR) method and is stored as a probability simplex that represents the probabilities that an RWR that starts at one node will end up at another node in equilibrium. A similar diffusion state between two nodes implies that the nodes are in similar positions within the network with respect to other nodes. Next, the node-specific diffusion state in individual networks are represented by two low-dimensional latent vectors: one that is shared across all networks and another that encodes the intrinsic topological property using multinomial logistic models. These shared low-dimensional node-specific latent vectors represent the homogeneous topological property across the network and can be used in other machine learning methods to derive further insights of the nodes. DCA has been applied to the functional analysis of genes (Cho et al., 2016) and drug-target interaction network (Luo et al., 2017).

While GeneMANIA and DCA integrates networks of features, similarity network fusion (SNF) (Wang et al., 2014) constructs a merged network between objects (e.g., biological samples) by combining multiple features types measured for each object. In particular, SNF first creates a network for the same set of samples from each data type, such as mRNA expression, DNA methylation, and microRNA expression. Then, it fuses these networks into one similarity network. The key idea of fusion is to update one network by utilizing two pieces of information: (a) the local affinity of the network and (b) the average similarity matrix of all the other networks. An iterative fusion process takes place, which increases the similarity between networks with each iteration until SNF achieves a final network by taking the average of all networks. In summary, SNF makes use of a network’s local structure, integrating both common and complementary information across networks. SNF has been applied to identify cancer subtypes and predict survival (Wang et al., 2014).

More network integration methods have been applied in genomics research in addition to the ones reviewed here, although they are often application specific and differ substantially from one another. For a more substantial review, we refer the readers to the review paper of Wani and Raza (2018). In general, network integration methods offer a simple and straightforward solution whereby similar nodes (e.g., genes and proteins) across multiple networks are integrated by merging different types of edges from multiple networks. Although simple, they are less efficient when it comes to preserving the relationships across multiple networks, particularly when the networks are heterogeneous and do not share the same biological mechanism.

## Unique Challenges of Microbiome Multi-Omics Network Analysis

Microbiome data science is often challenged by various statistical properties of microbiome data, including its compositionality, heterogeneity, and sparsity. These properties impact how statistical methods are applied to microbiome data and require careful consideration to ensure appropriate analysis. In this section, we discuss these various properties and how they impact the application of the approaches described in Section “Review of Available Network-Based Procedures” to microbiome data, especially with respect to microbiome multi-omics data integration. Our hope is that this discussion helps readers identify opportunities to transform microbiome multi-omics network analysis.

### Compositionality

**Figure 1:**
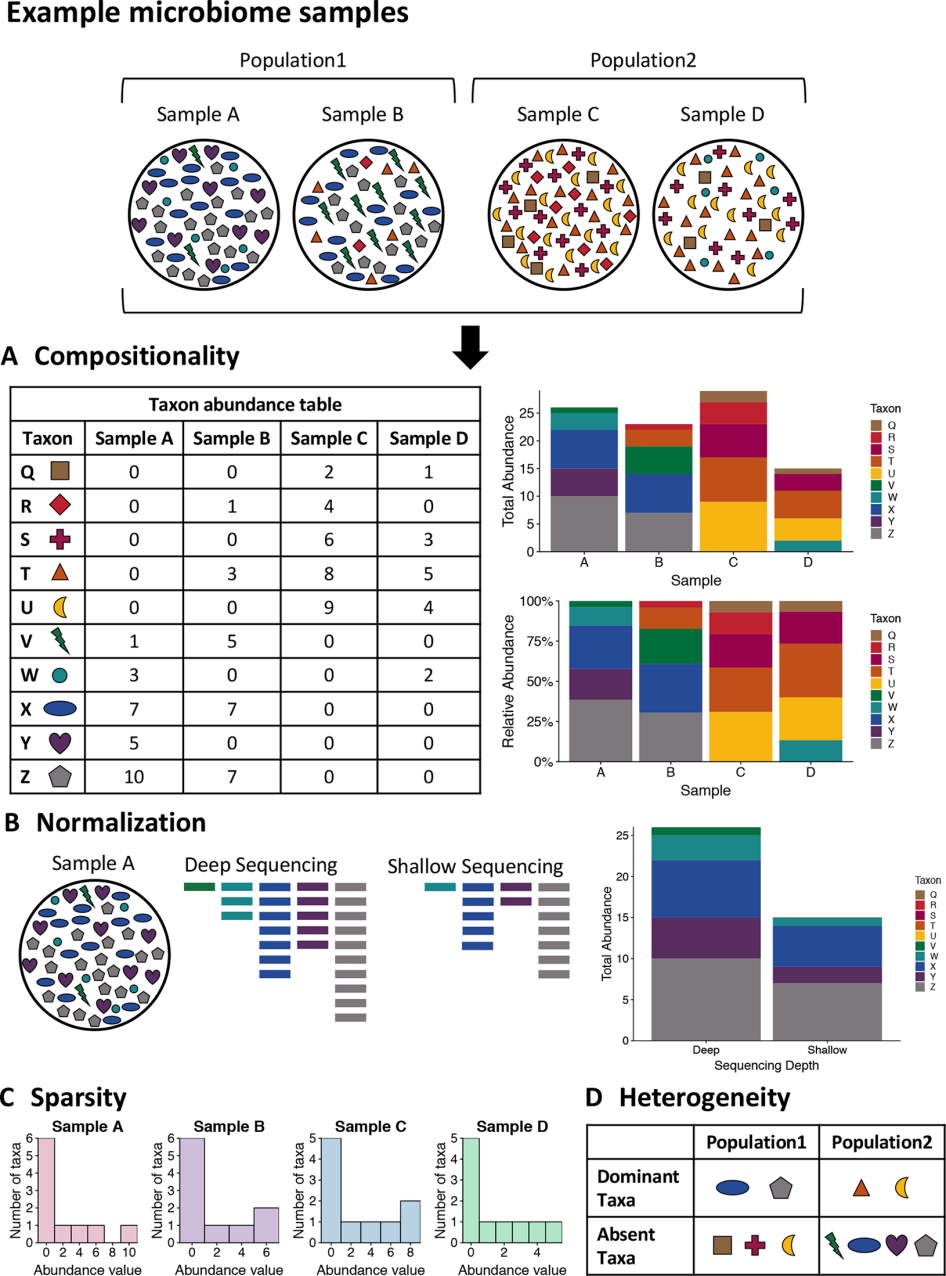
Visualizing the unique challenges of microbiome data. A mock set of bacterial samples from two populations where each colored shape is a bacterial taxon. **(A)** Compositionality. The taxon abundance table depicts the count of each observed taxon in each sample. When sequencing microbiome samples, the resulting counts of taxa are not representative of the actual taxa counts in the sample due to constraints of sequencing. Due to this, relative abundances are generally used in analysis of microbiome data. The bar plots illustrate the difference in community representation between raw counts (top) and relative abundances (bottom). **(B)** Normalization. Due to the constraints of sequencing, the overall sequencing depth of a sample can impact the results. For example, shallow sequencing may miss rare taxa such as the green taxon V in the example sample A that is present in low abundance in the community. **(C)** Sparsity. Microbiome data are often very sparse, where most observations are zero. This is illustrated by the histogram of taxa counts for each sample where most counts are zero and there are few taxa with high counts. This can also be seen in the table for part A, where many entries are zero. **(D)** Heterogeneity. The table summarizes the taxonomic heterogeneity in the mock dataset between the two populations. Each sample has a unique taxonomic composition, but there are also population specific signatures. The samples in each population are dominated by a few taxa, and these dominant taxa are different for the two populations. Additionally, there are taxa that are highly abundant in one sample and absent from the rest, such as the purple taxon Y in sample A.

One of the unique characteristics of microbiome data is its compositionality. Microbiome data are often presented as the abundances of different microbial taxa contained in a microbial community. However, microbiome data only carry information about the relative abundances of the taxa instead of their true abundances. This is because the total sequence count of all taxa for each sample, known as the sequencing depth of the sample, is an experimental technicality imposed by the sequencing instrument and bears no biological relevance. Therefore, the abundance count of a taxon in a sample only reflects the relative abundance of the taxon compared against all other taxa, rather than the absolute count of molecules in the underlying community attributable to the taxon. As a result, these data exist under an arbitrary sum constraint and are thus referred to as compositional data. This feature is also visualized in **Figure 1A**.

When modeling compositional data, it is important to account for the fact that the sum is uninformative about (i.e., ancillary for) the parameters of interest, and therefore, it may be desirable to consider the conditional distribution of the data regarding the sequencing depths as pre-fixed quantities. For example, a common strategy to acknowledge compositionality of microbiome data is to convert the abundance count of each taxon into proportions or relative abundances that sum up to one for each sample. A consequence of the sum constraint is that the features will tend to be negatively correlated even if the underlying (unobserved) true abundances are independent.

The traditional marginal correlation analysis methods in Section “Marginal Correlation Analysis” such as Pearson’s, Spearman’s, and Kendall’s correlations do not consider microbiome data compositionality. The key issue is that there exists a constraint on the correlations between one taxon and all other taxa due to the compositionality of the data, which can yield spurious inferences of interaction. For example, for any given taxon, its Pearson’s correlation coefficients with the other taxa always sum up to −1, regardless of how this taxon interacts with the rest of the microbiome. Recently, new methods have been proposed to account for data compositionality when constructing microbial networks. For example, SparCC (Friedman and Alm, 2012) employs a log-ratio transformation for every pair of taxa being correlated to remove compositionality: the ratio of the abundances of two taxa is independent of which other taxa are included in the analysis, a property termed subcompositional coherence. SparCC also uses an iterative algorithm that identifies the pair of taxa with the strongest correlation in each step and terminates iterations when a relatively sparse network structure is obtained. More recently, CCLasso (Fang et al., 2015) and REBACCA (Ban et al., 2015) use global optimization procedures that estimate the correlation network of all species while imposing an explicit constraint caused by the compositionality of the data and a sparsity constraint on the network. While this approach is effective at controlling for data compositionality, these methods are only designed to reconstruct taxon-taxon interaction networks. To the best of our knowledge, we are unaware of approaches that consider compositionality when constructing microbiome multi-omics networks.

The compositionality of microbiome data has also been considered in methods based on graphical models (Section “Methods Based on Graphical Models”). Given that the major goal of graphical modeling is to infer microbial interactions through the estimation of the inverse covariance matrix between species, it is harder to correct for data compositionality as compared to marginal correlation analysis. The unique challenge here is that the sum constraint in compositional data induces linear dependency between features and thus gives rise to a degenerate covariance matrix, meaning that the inverse covariance matrix does not exist. To overcome this challenge, Kurtz et al. (2015) proposed a method called SPIEC-EASI that first converts raw counts into relative abundances, i.e., the proportions of each taxon’s abundance within a sample, and then uses the centered log-ratio transformation on the relative abundances. They further argue that the covariance matrix of the transformed relative abundances is a good approximation to that of the log-transformed raw counts. SPIEC-EASI uses both neighborhood selection (Meinshausen and Bühlmann, 2006) and graphical lasso (Friedman et al., 2008) to infer a sparse inverse covariance matrix for a network. In addition, Yang et al. (2017) proposed a method called mLDM that uses a hierarchical Bayesian model (lognormal-Dirichlet-multinomial) on the compositional counts and then estimates a sparse inverse covariance matrix between the species through maximizing the L1 penalized posterior distribution.

Compositionality is also important to consider in regression-based methods. In Section “Review of Available Network-Based Procedures”, we reviewed several regression methods to construct biological networks. To apply these methods to integrate microbiome data and another data type, it is possible to use microbiome data as either predictors or responses. Therefore, we discuss these two situations separately. In the case that microbiome data are used as predictors, there are two major challenges: the high dimensionality of the data and a sum constraint on the predictors imposed by the compositional nature of the data. Lin et al. (2014) proposed an L1 regularization method for the linear log-contrast model that meets these unique challenges of compositional data to study the association between the microbial compositions and the response variable. Moreover, Shi et al. (2016) extended the previous method to consider the subcompositions of taxa, i.e., the composition of taxa that belong to a given higher level taxonomic rank, and studied whether the observed subcompositions are associated with the response variable. On the other hand, if microbiome data are used as responses, it is essential to incorporate an appropriate distribution in the model to reflect the compositionality. For example, Chen and Li (2013) applied the Dirichlet-multinomial regression to investigate the association between microbiome composition and environmental covariates. Furthermore, Xia et al. (2013) proposed to use the logistic normal multinomial regression model to link covariates with taxonomic counts, given that the logistic normal distribution has a more flexible covariance structure than the Dirichlet distribution. The mLDM method (Yang et al., 2017) also investigates the association between the taxonomic counts and the environmental factors in their lognormal-Dirichlet-multinomial model.

As mentioned above, many network analysis methods have been proposed to consider the compositionality of the microbiome data. However, very few of them have been applied for network analyses that integrate multi-omics data alongside microbiome measures. We anticipate that this will be an active research area in the near future. Moreover, technological developments in microbiome data science, including the estimation of absolute cellular abundances from microbiome sequence data (Vandeputte et al., 2017) may help offset the need to correct for data compositionality when reconstructing microbiome networks.

### Normalization

Similar to many other omics data, microbiome data can exhibit strong heterogeneity from one study to another or from one biological sample to another even in the same study. For example, microbiome data may be collected from different geographic populations and they may have very different taxonomic distributions (He et al., 2018). In addition, varying data generation and processing procedures for microbiome data can also lead to heterogeneity across studies. For example, different sequencing technologies will result in different sequence lengths across studies, which can impact the discovery of taxa. Moreover, different studies may apply different data processing procedures (e.g., how sequences are assigned to taxonomic units or phylotypes) that may impact the distribution of taxa across studies.

One unique heterogeneity between studies or between samples in microbiome data is the variation of sequencing depths, as visualized in **Figure 1B**. Sequencing depth, the total count of sequences generated across all taxa for a biological sample, is an experimental technicality and often varies considerably across samples in a microbiome sequencing experiment. Like other omics data, normalization is an important and often first analytical step. The traditional approaches for normalizing microbiome data is either to transform count-based measures of taxa into relative abundances (i.e., proportions) of the taxa or to rarefy the counts, i.e., subsampling without replacement from each sample such that all samples have the same number of total counts across taxa. In addition, alternative normalization methods using other criteria are also used in the microbiome research community, including upper quantile normalization (Bullard et al., 2010), CSS normalization (Paulson et al., 2013), variance stabilizing transformation (Love et al., 2014), and trimmed mean of M-values normalization (Robinson et al., 2009; McCarthy et al., 2012). Most of these alternative normalization methods are borrowed from the techniques for RNA-seq data analysis. While these alternative methods are advocated in studies that focused on differential abundance testing, the traditional approaches of proportion- and rarefaction-based normalization provide more accurate community-level comparisons (McKnight et al., 2018).

Studies have also assessed the influence of sequencing depth on the quality of microbiome data. For example, Jovel et al. (2016) measured the minimum sequencing depth that can still provide a consistent taxonomic classification by randomly sampling from a sequencing library with different depths, while Nayfach et al. (2015) conducted a similar analysis for the functional annotation of metagenomes. Zaheer et al. (2018) evaluated the impact of sequencing depth on the characterization of the microbiome and resistome and indicated that the relative proportions of sequence assignments remained fairly constant regardless of depth. Although these studies show that taxonomic and functional annotation is fairly stable regardless of the sequencing depth, McMurdie and Holmes (2014) argued that current practice in the normalization of microbiome count data is inefficient in the statistical sense. One key issue with rarefaction is that while it maintains the mean of the taxonomic proportions it ignores the variation of the proportions. For example, two equal proportions of an OTU in two samples can have unequal variances due to the different sequencing depths between the two samples. This problem of unequal variances is called “heteroscedasticity” and is not accounted for during typical rarefaction approaches. Heteroscedasticity could impact downstream analysis such as differential abundance analysis and construction of microbial networks.

In Section “Compositionality”, we reviewed statistical models such as Dirichlet-multinomial regression (Chen and Li, 2013), logistic normal multinomial regression (Xia et al., 2013), and mLDM (Yang et al., 2017). These models not only consider the compositionality of microbiome data but also take the heteroscedasticity into account because the sequencing depth is explicitly modeled in the multinomial distribution. However, most of the above methods are applied to identify the association between the taxonomic composition and the environmental factors. While these models are potentially applicable to network analyses that integrate microbiome and other omics data, further investigations are warranted, especially considering the scale of the dimensionality of multi-omics data.

### Sparsity

Taxonomic abundance data are typically sparse in nature, meaning that a high proportion of the counts are zeros (Paulson et al., 2013). This feature of microbiome data frequently poses challenges to common statistical methods, and tailored techniques are often required to properly analyze microbiome data and to integrate them with other omics data. For example, due to the compositionality of microbiome data (see Section “Compositionality”), many statistical methods utilize transformations that involve taking logarithms on the counts or ratios between them. However, zero counts cause a technical problem for these transformations. To circumvent this issue, a widely used strategy is to add a small constant to all count measures, known as a pseudo-count (Kurtz et al., 2015; Mandal et al., 2015), or to replace the zeros by an estimated value (Palarea-Albaladejo and Martín-Fernández, 2015; Gloor et al., 2016). Some recent work has studied the problem of how to best choose the pseudo-count and how to find the estimated value (Martín-Fernández et al., 2003; Martín-Ferńandez et al., 2011; Martín-Fernández et al., 2015). However, more research is needed to determine how these techniques impact integrative network estimation for microbiome multi-omics data.

The sparsity of microbiome data also challenges modeling. The excess zeros, coupled with a high frequency of a very low number of observations per taxon, results in a heavily skewed distribution of taxon counts across samples, with a large point mass at zero and a long right tail. This is also visualized *via* a mock dataset in **Figure 1C**. Consequently, network estimation methods that work well for continuous data, including those assuming that the counts follow a Gaussian distribution such as graphical lasso, may not work well when directly applied to such data because of poor model fit. Nonparametric correlation measures such as Spearman’s rank correlation and Kendall’s tau can be used to avoid an assumption of normality and tackle highly skewed data. However, the power of such methods may deteriorate when data measures distribute with a point mass at zero, as this mass of zeros leads to a large number of ties that complicate rank-based measures of correlation (Huson, 2007). In addition, agglomeration of taxon measures into higher order taxonomic groups may reduce the effects of sparsity and improve alignment between the observed data distributions and model assumptions. However, such agglomerative procedures can erode resolution of specific taxonomic units that manifest important and nuanced relationships with other study covariates.

In recent years, a variety of probability models have been developed for microbiome count data. The Poisson or negative binomial distributions have been useful for analyzing count data from other types of sequencing studies, such as transcriptomic studies using RNA sequencing. However, microbiome data often—though not always—exhibit more zeros and heavier skewness than expected from these models. To this end, zero-inflated models (Sharpton et al., 2017) and hurdle models (Hu et al., 2011) have been proposed. For example, the zero-inflated Poisson distribution considers a mixture of a Poisson distribution and a probability mass at zero to account for the large frequency of zeros in microbiome data (Xu et al., 2015). However, most of these methods focus on modeling the marginal distribution of a single taxon at a time and are not directly applicable to the joint modeling of multiple taxa and therefore cannot be used for microbial network estimation.

Another type of models used for microbiome count data is the Dirichlet-multinomial model and its zero-inflated versions. It has been used in a number of methods to model the multivariate distribution of the counts of a collection of taxa (Holmes et al., 2012; Chen and Li, 2013; Tang and Chen, 2018). However, a criticism of these methods is that the Dirichlet-multinomial distribution imposes a negative correlation between the abundances of any given pair of taxa. This inflexibility in the correlation structure makes such methods particularly problematic when used to infer the interaction between taxa. A promising approach to addressing this pitfall is to consider a hierarchical model where the conditional distribution of the observed counts is modeled by a multivariate count distribution such as multinomial distribution or Dirichlet-multinomial distribution, whose parameters are linked to a multivariate continuous distribution, such as multivariate normal distribution, that allows a flexible and realistic correlation structure (Xia et al., 2013; Yang et al., 2017).

Despite the success of the aforementioned models for microbiome count data, their use has for the most part been limited to differential abundance analysis, where the abundance of individual or groups of taxa is associated with an environmental factor of interest. Further work is needed to explore their applicability to multi-omics data and integrative network analysis. We see it as a great research opportunity to combine these models with cutting edge multi-omics network estimation methods to make the latter more appropriate for microbiome studies.

### Heterogeneity

Related to the issue of sparsity is the heterogeneity exhibited in studies that survey the composition of microbial communities. The composition of microbial communities often varies tremendously across hosts and environments. For example, it is not uncommon to observe that a taxon that is relatively abundant in one person’s gut while being completely absent in another’s; for a given taxon, it is often the case that only a proportion of the samples have nonzero abundance. While the number of observed taxa from the entire data set may be large, the microbiota in any given sample tend to be dominated by only a relatively small number of taxa with high abundance, with the rest of the taxa having zero and very low counts. Moreover, the set of dominant taxa can vary drastically from individual to individual. We call the above phenomena taxonomic heterogeneity, as visualized in **Figure 1D**. It results in a unique characteristic of microbiome data sets that features (i.e., taxa) present in all samples are rare and those present in a small proportion of samples prevail. This is in contrast to most other types of omics data such as transcriptomic data, where the majority of genes are expected to have nonzero expression levels in all samples.

Different approaches have been applied to account for taxonomic heterogeneity when measuring the interaction between two microbial taxa or between a taxon and another biological feature (e.g., a metabolite). The most commonly used strategy is to include the data from all biological samples, regardless of whether the taxon of interest is present or not. An alternative strategy is to exclude the samples in which the given taxon is not present and only consider those abundance data that are nonzero for the taxon. A third strategy focuses on the dichotomous outcome of whether a taxon is present or absent in individual samples, while ignoring the actual abundance (Mainali et al., 2017; Albayrak et al., 2018). The first approach regards a sample where a taxon is absent as having “zero abundance” of the taxon, which is only quantitatively, but not qualitatively, different from a sample where the abundance of the taxon is very low. This approach’s main advantage is that no information is discarded from the data, whereas the latter two approaches each discard part of the data. Most methods using the first approach assume that, if a biological interaction exists between a microbial taxon T and another feature M (e.g., a metabolite), the feature M is associated with the abundance of T in the same way that it is associated with the occurrence of T in a community. However, the biological process in which M is involved in the introduction or establishment of T may in theory be very different from the one in which M impacts its abundance. For example, M may promote the growth of T in a person’s gut microbiome only if it already contains T. It is also possible that elevated levels of M are associated with increasing a person’s chance of exposure to T and consequently its presence in the gut, but do not affect its abundance. For these types of relationships, the latter two strategies may have merits.

In addition to taxonomic heterogeneity, functional heterogeneity is another feature of microbiome data that challenges statistical methods for network inference. Most current methods for microbial network estimation, such as those by Kurtz et al. (2015) and Yang et al. (2017), assume that there exists a common microbial network underlying all samples in the data. However, the interaction between two microbial taxa or between a taxon and another type of feature may be context dependent and may vary from sample to sample. For example, the interaction between taxa in the human gut may depend upon the enterotypic context of the individual’s gut microbiome. Recent statistical developments have been made on the joint estimation of multiple graphical models, which assumes the samples are from several known subpopulations (e.g., corresponding to several biological conditions) and allows a different network to be inferred for each subgroup (Chun et al., 2015; Lin et al., 2017). In addition, some emergent methods have been applied to genomic data to allow network heterogeneity among all samples, between or within biological conditions. For example, Luo and Wei (2018) developed a nonparametric Bayesian method to estimate dynamic transcription factor networks by borrowing information across biological conditions and meanwhile allowing heterogeneity across samples. Another example is mixGlasso (Städler et al., 2017), a latent variable extension of graphical lasso, which uses a mixture model to allow samples to be clustered into groups that can have different networks. Despite these recent statistical developments, methods have not been established to address the unique needs of microbiome data analysis and for the purpose of integrating microbiome multi-omics data.

## Discussion

This review focuses on statistical network analysis methods that have been applied or have great potential to be applied to multi-omics integration of microbiome data. Therefore, this review does not cover some of the other analytical methods and tools that are either not directly relevant to statistical network analysis or not specific to microbiome data but are still applicable to general multi-omics integration. For these more general methods and tools, we refer the readers to the following review papers. Bersanelli et al. (2016) categorized various data integration methods into four classes according to whether they are Bayesian and whether they are network-based, and they reviewed each class of methods focusing on their mathematical and methodological aspects. Li et al. (2018) provided a comprehensive review on omics and clinical data integration techniques from a machine learning perspective. Huang et al. (2017) separately reviewed unsupervised, supervised, and semisupervised data integration tools and their applications to predicting patient survival. Zeng and Lumley (2018) reviewed the traditional statistical methods of exploratory and supervised learning as well as their variations tailored to multi-omics studies. Mirza et al. (2019) discussed state-of-the-art machine learning-based approaches for tackling five specific computational challenges associated with integrative analysis: curse of dimensionality, data heterogeneity, missing data, class imbalance, and scalability issues.

While our review focuses on data analysis, it is important to note that study design and data collection can impact data integration-based investigations. For example, in a multi-omics study, it is rarely the case that researchers are able to collect a complete data set in the sense that all feature types are measured for all samples. This incomplete coverage of samples can dramatically reduce the set of samples subject to integration. In a longitudinal multi-omics study of the gut microbial ecosystem in inflammatory bowel diseases (Lloyd-Price et al., 2009), 132 participants were followed for one year and their stool samples were collected every two weeks, resulting in 1,785 stool samples. However, given the difficulty of collecting all feature types (for example, metagenomics, metatranscriptomics, proteomics, metabolomics, etc.) at each timepoint, the final data include only 305 samples that yielded all stool-derived feature types, whereas 791 samples offered paired metagenomic and metatranscriptomic data. As exemplified in this study, to derive networks depicting the relationships between certain pairs of feature types, one may need to rely on separate sets of samples for the two feature types. This strategy, compared with one in which paired multi-omic data are available on a common set of samples, would impact the accuracy and interpretation of the resulting networks. In addition to the above practical issue of missing data, considerations of study design can impact integration, such as whether the samples were collected longitudinally or cross-sectionally. Given that this is a very broad topic, we refer readers to additional review papers (Franzosa et al., 2015; Buescher and Driggers, 2016; Haas et al., 2017; Hasin et al., 2017) for more detailed discussions about how study design impacts multi-omics investigations.

The recent work by the Integrative Human Microbiome Project (iHMP, https://hmpdacc.org/ihmp/) exemplifies the power and promise of microbiome multi-omic data integration. As the second phase of the NIH Human Microbiome Project, iHMP aimed to link interactions between humans and their microbiomes to health-related outcomes by analyzing data sets on microbiome and host activities in longitudinal studies of disease-specific cohorts (Integrative HMP (iHMP) Research Network Consortium 2014; Integrative HMP (iHMP) Research Network Consortium 2019). Fortunately for the research community, the iHMP has made these measures publicly available as downloadable datasets that can serve as resources to test and evaluate new models, methods, and analyses, including the network methods reviewed in this paper. In fact, many of the individual studies conducted as part of iHMP have applied and/or developed network-based methods for integrating multi-omics data. For example, Lloyd-Price et al. (2019) applied integrative analysis to identify microbial, biochemical, and host factors central to the functional dysbiosis in the gut microbiome during inflammatory bowel disease activity. They constructed networks for associations of features from 10 feature types: metagenomic species, species-level transcription ratios, functional profiles at the Enzyme Commission level (metagenomes, metatranscriptomes, and proteomes), metabolites, host transcription (rectal and ileal separately), serology, and fecal calprotectin. In particular, they used mixed-effects regression models (which belong to the regression-based methods discussed in Section “Regression-Based Methods”) to remove subject-specific random effects and covariate effects from each feature type, and then applied Spearman correlation (which belong to the marginal correlation analysis methods discussed in Section “Marginal Correlation Analysis”) to the resulting residuals to construct cross-feature type interactions.

We conclude this review with some final thoughts about microbiome multi-omics network analysis. Integrative network analysis holds great potential to resolve how microbes interact among themselves and with their environment. However, the application of such analyses to microbiome data remains nascent, and the requisite analytical tools have only begun to emerge. Fortunately, a growing number of statistical methods have been developed in the fields of network estimation and multi-omics data analysis, which provide a promising pool of ideas and methodologies to potentially borrow from. However, when applying these existing tools to microbiome multi-omics network inference, it is important to consider the limitations of the underlying methodologies and their applicability to microbiome studies. In particular, the unique features of microbiome data present pressing statistical challenges and often call for tailored computational tools. A thorough understanding of the unmet statistical needs and specific properties of microbiome data is critical to the innovation of efficient, robust, and scalable network inference methodologies suitable for microbiome multi-omics network inference. Meanwhile, awareness of the analytical challenges associated with microbiome data can facilitate the development of new study designs and technologies that have the potential to mediate some of the major limitations currently hindering microbiome data analytics. An emerging example is the coupling of 16S data with measures of the total abundance of microorganisms in a sample, which is a possible way of circumventing the compositionality constraint in microbiome data. Going forward, joint statistical, scientific, and technological efforts will help promote the application of multi-omics network analysis to solve pressing problems in microbiome science.

## Author Contributions

DJ, TS, and YJ led and conducted the review. CA, CH, MM, and CT contributed equally to the review and wrote the first draft of sections of the manuscript. DJ, TS, and YJ wrote the first draft of sections of the manuscript and contributed to the manuscript revision. All authors read and approved the final version.

## Funding

Research reported in this publication was supported by the National Institute of General Medical Sciences of the National Institutes of Health under award number R01GM126549. The content is solely the responsibility of the authors and does not necessarily represent the official views of the National Institutes of Health.

## Conflict of Interest

The authors declare that the research was conducted in the absence of any commercial or financial relationships that could be construed as a potential conflict of interest.
